# Supplementary material for: Causal Relationship between Aging and Anorexia Nervosa: A White-Matter-Microstructure-Mediated Mendelian Randomization Analysis
Source: Biomedicines. 2024 Aug 16;12(8):1874. doi: 10.3390/biomedicines12081874 (PMC11351342; doi:10.3390/biomedicines12081874)
Supplement: Supplementary file 1 [file biomedicines-12-01874-s001.zip › Supplementary Figure/Figure S4/Singe SNP plot of MR analysis for TL to ubm-b-1486.pdf]

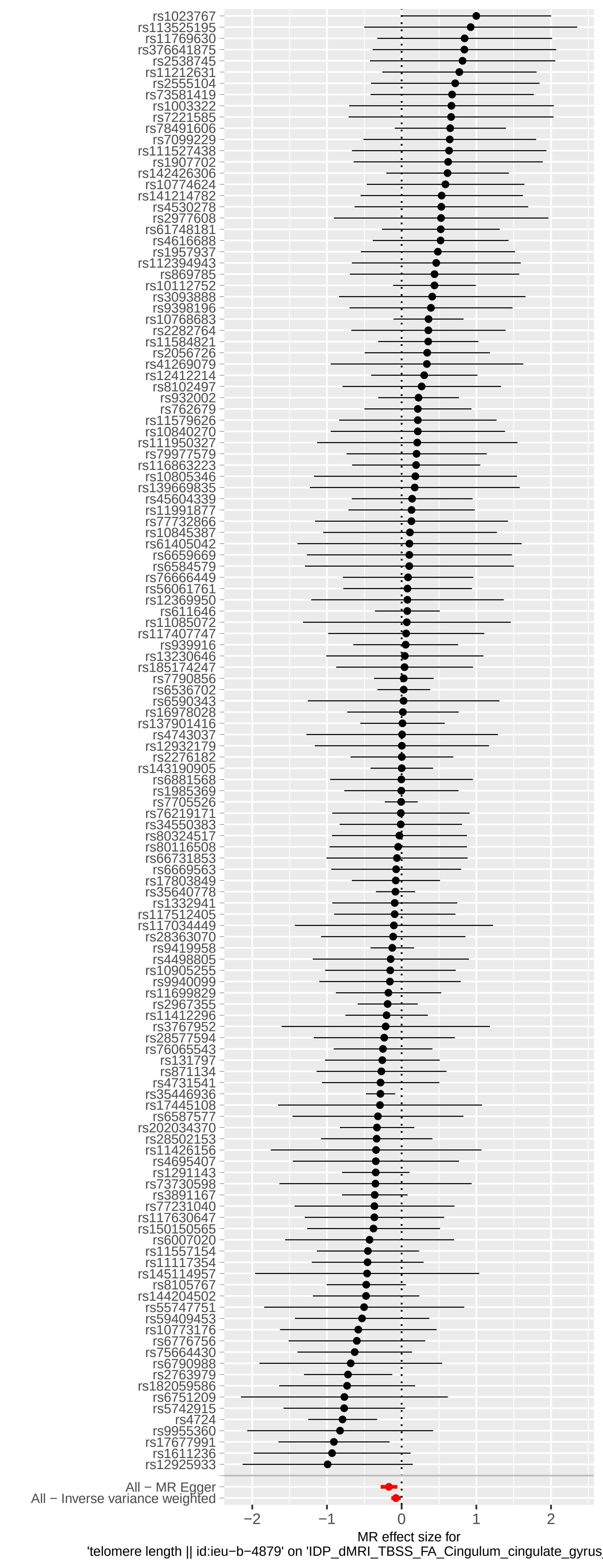

rs1023767
rs113525195
rs11769630
rs376641875
rs2538745
rs11212631
rs2555104
rs73581419
rs1003322
rs7221585
rs78491606
rs7099229
rs111527438
rs1907702
rs142426306
rs10774624
rs141214782
rs4530278
rs2977608
rs61748181
rs4616688
rs1957937
rs112394943
rs869785
rs10112752
rs3093888
rs9398196
rs10768683
rs2282764
rs11584821
rs2056726
rs41269079
rs12412214
rs8102497
rs932002
rs762679
rs11579626
rs10840270
rs111950327
rs79977579
rs116863223
rs10805346
rs139669835
rs45604339
rs11991877
rs77732866
rs10845387
rs61405042
rs6659669
rs6584579
rs76666449
rs56061761
rs12369950
rs611646
rs11085072
rs117407747
rs939916
rs13230646
rs185174247
rs7790856
rs6536702
rs6590343
rs16978028
rs137901416
rs4743037
rs12932179
rs2276182
rs143190905
rs6881568
rs1985369
rs7705526
rs76219171
rs34550383
rs80324517
rs80116508
rs66731853
rs6669563
rs17803849
rs35640778
rs1332941
rs117512405
rs117034449
rs28363070
rs9419958
rs4498805
rs10905255
rs9940099
rs11699829
rs2967355
rs11412296
rs3767952
rs28577594
rs76065543
rs131797
rs871134
rs4731541
rs35446936
rs17445108
rs6587577
rs202034370
rs28502153
rs11426156
rs4695407
rs1291143
rs73730598
rs3891167
rs77231040
rs117630647
rs150150565
rs6007020
rs11557154
rs11117354
rs145114957
rs8105767
rs144204502
rs55747751
rs59409453
rs10773176
rs6776756
rs75664430
rs6790988
rs2763979
rs182059586
rs6751209
rs5742915
rs4724
rs9955360
rs17677991
rs1611236
rs12925933
All − MR Egger
All − Inverse variance weighted
−2
−1
0
1
2
MR effect size for
'telomere length || id:ieu−b−4879' on 'IDP_dMRI_TBSS_FA_Cingulum_cingulate_gyrus_
